# Challenges to Promote Sustainability in Urban Agriculture Models: A Review

**DOI:** 10.3390/ijerph20032110

**Published:** 2023-01-24

**Authors:** Luiza Vigne Bennedetti, Paulo Antônio de Almeida Sinisgalli, Maurício Lamano Ferreira, Fabiano Lemes de Oliveira

**Affiliations:** 1 Environmental Science Graduate Program, University of São Paulo, São Paulo 04021-001, Brazil; 2 Geoenvironmental Analysis Master Program, University of Guarulhos, Guarulhos 07023-070, Brazil; 3 Department of Architecture and Urban Studies, Politecnico di Milano, 20133 Milan, Italy

**Keywords:** urban food production, urban agriculture, socio-environmental models, sustainable development, public health policies

## Abstract

Urban agriculture (UA) can be used as an action to promote sustainability in cities and inform public health policies for urban populations. Despite this growing recognition, its implementation still presents challenges in countries in the Global North and Global South. Background: In this context, this systematic review aims to identify the development of frameworks for the implementation of UA as a sustainable action and its main opportunities and shortcomings in meeting urban socio-environmental demands. Methods: In this review, using the PRISMA protocol, we evaluated 26 studies on the interplay between UA and sustainability surveyed on the Web of Science to provide an overview of the state of the art. Conclusions: In summary, it was possible to identify many key challenges in UA adoption, which regard air and soil contamination, availability of green areas, layout of urban infrastructure, food distribution, among others. Due to numerous socio-economic and environmental contextual factors in cities, especially when comparing realities of the Global North and Global South, there is a need to develop a model that can be adaptable to these different contexts. Thus, it is recognized that the concept of sustainability does not present a universal understanding and that in its search it could be argued that one of the most important gaps is still to address social issues in relation to environmental ones.

## 1. Introduction

Urban agriculture (UA) movements gained strength from the late 1970s onwards in response to rising poverty and food prices [1]. Some authors considered it a phenomenon able to enhance the resilience of communities in moments of socio-economic crises [2,3]. Although its impact is still poorly understood, it is estimated that at least 200 million urban producers work in cities worldwide, providing food for about 800 million people [4].

In addition to food and nutritional security, authors argue that UA can provide provisioning, regulation, support, and cultural ecosystem services (ES) [5], as well as performing the integration of values such as dignity, equity, inclusion, and justice [6]. Examples of ES provided by UA include, besides food production, water and climate regulation, pollination, biodiversity habitats, among others [7,8].

Cities have already been recognized as “engines” for sustainable development and climate mitigation [9,10,11], with those from developing countries potentially being driving forces for positive change regarding climate adaptation [12]. Based on land-use policies, for example, cities can change their urban metabolisms so that they become more circular and ecological, reducing thus waste and pressure upon the environment [13,14]. In this context, it is recognized that UA deserves a strategic approach in city planning and food security policies since it can act as an instrument for alleviating urban challenges.

As such, many authors argue that UA can be implemented to promote sustainability in cities [7]. However, at the same time, studies highlight the need to implement policies that promote UA while not stimulating a greater demand for resources in the urban environment [15,16]. Dobson et al., for instance, argue that only through a circular approach can the sustainability of UA be truly reached [17].

Yet, a clear and specific definition of economic strategies for UA mainstreaming is still lacking [18]. In this regard, public contribution is limited as this sphere plays a crucial role in creating political frameworks to facilitate access to land, financing, and information. Increasing urban resilience requires collaborative thinking and cooperation between governments, academy, and communities, as well as creative and context-specific solutions based on a comprehensive assessment of local conditions [19].

Although UA’s potential to help meet sustainability indicators is recognized, it is essential to have clarity on, after all, what is intended for sustainability and for whom. In this light, this article proposes that for an UA initiative to succeed and be truly sustainable, it must be customized to its socio-environmental context. In this regard, some studies seek to point out an analysis model for UA; however, it is still a challenge to align social and environmental issues. Therefore, the research question that guides this work refers to the identification of opportunities and gaps to situate the UA’s contribution to urban sustainability. The remaining of the article is structured as follows: the next section discusses the concept of sustainability. The third explores examples of how UA is able to help meet sustainability indicators. The fourth discusses the governance challenge. The fifth points out the characteristics of initiatives in the Global North and South. Finally, the need to develop models to evaluate UA is discussed.

## 2. Sustainability

The concept of sustainability began to be outlined in the 1980s based on the increasing acknowledgement of planetary environmental degradation [20]. It then made its way into the agendas of important institutions, such as the UN. The Brundtland Report defined the concept of sustainable development as “one that meets the needs of the present without compromising the ability of future generations” [21]. In this context, an integrated approach to the concept of sustainability represents a system encompassing different levels that include social, environmental, and economic aspects [20].

In time, the concept of ecological design came to be associated with sustainable development. Such convergence triggered new ideas and technologies in different fields of activity. Topics such as renewable energy, alternative materials, recycling, agroecological food and environmental footprint were being absorbed by the market and political agendas [22,23,24,25].

In opening up the possibility of a better relationship between development and the environment, such innovations led the way to the emergence of a green economy, with sustainability incorporated as a capitalist value. This, on the one hand, paved the way for political incentives for ‘green’ businesses [26], but, on the other hand, also evidenced a weak recognition of the needs and identities of historically marginalized groups [27] and the phenomenon of greenwashing.

At the beginning of the 2000s it was emphasized that the present distance between the poor and the rich is a limiting factor for reaching and maintaining global stabilities, and the preservation of natural resources [28]. Thus, it is understood that poverty and the environmental crisis are related [29].

In the last 20 years, there has been a significant development of new tools and approaches that incorporate ecological considerations in a range of fields; however, their uptake is still taking slow steps.

## 3. Materials and Methods

We used the PRISMA 2020 framework [30] to survey and analyze the studies that combine sustainability and UA. The Web of Science database was used to collect documents. Firstly, the inclusion and exclusion criteria were set. The keywords “urban agriculture” and “sustainability” were defined to construct the search expression. In this step, only complete articles available online, written in English, and relevant to the study were included. Finally, the exclusion criteria were defined: books, conference reports, and grey literature.

In the second stage, we conducted a systematic literature review by following the PRISMA protocol. The search expression (title or publication title contains the terms (“urban agriculture”) AND (“sustainability”) was used. The articles were organized in two groups: most cited publications in the last 10 years (2012–2022) and publications from the last two years (2021–2022). The aim was to better explore the most relevant and recent trends within the proposed theme. The search took place in March 2022. The initial sample contained 129 documents. After removing duplicates and those meeting the exclusion criteria, a sample of 125 articles was identified. Abstracts were then read by one of the authors and discussed with others in order to identify articles relevant to answering the research question. See Table 1.

**Figure 1:**
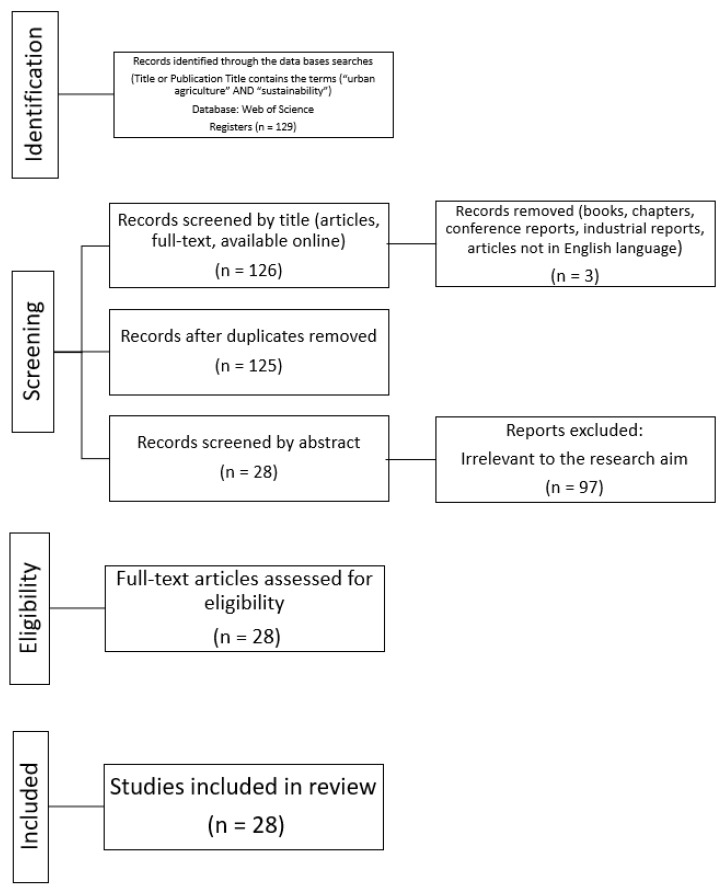
The PRISMA selection process of relevant literature.

After the above step, 28 articles were selected (listed in Table 2) to make the final sample. The literature screening and selection process is illustrated in Figure 1 [30].

The results from these studies relating to present potential gaps or opportunities for the development of UA as a sustainable measure were identified, as seen in Table 2. For that, ‘gaps’ were defined as hiatus that still need to be better understood in order to find solutions for successful UA implementation. ‘Opportunities’ were considered when articles explicitly identified the success potentials of the UA. This strategy was adopted as a way to better understand the state of the art of the UA.

## 4. Results and Discussion

After applying the inclusion and exclusion criteria, this review included 28 articles in the English language published in scientific journals. The articles selected are distributed across 15 journals and four continents, Europe being the one with the highest number of publications. A total of 11 studies were categorized with “opportunities outcomes”, 13 studies with “gap outcomes”, and four studies with both “opportunities and gap outcomes” (Table 2).

### 4.1. Urban Agriculture as a Sustainable Action

Currently, urban centers are responsible for consuming two-thirds of the energy in the world and for up to 70% of global greenhouse gases (GHG) emissions [31]. In this context, as urban areas lost their ability to self-supply [8], importing food from local producers became crucial. A recent publication pointed out that, in 2018, total world emissions from food transport were 511 Mt CO_2_, with a global increase of 80% of these emissions since 1990 [32]. This transport implies the loss of both quantity and quality of food and is considered the main contributor to food waste by some authors [33]. Global data on food loss that occurs between harvesting and reaching the final consumer, including transport, is estimated at 14% of the total loss [34]. In this context, UA has been enthusiastically defended as actions that can help address these challenges. [35].

Covering most empty urban areas with edible green could be a new ecological achievement as such interventions could reduce urban pollution, mitigate, and adapt to climate change by increasing carbon sequestration, water infiltration and retention, and controlling the urban heat island effect [36,37]. Green roofs and vertical walls can also reduce heating and cooling while improving air quality and contributing to increased biodiversity and ES [37].

UA is compatible with the proposals of the circular economy movement [38], where the optimization of processes based on durable, recyclable, and renewable resources is prioritized. Thus, models that integrate food production with organic waste management have a high potential to encourage the reuse of nutrients and the development of by-products. In the United States, it is estimated that around 28% of household organic waste could be reused for food production [39]. Similarly, in Havana, organoponics was implemented using organic waste from different sources, including domestic waste [40]. In addition, given the water resource management crisis in many urban areas, UA initiatives must be jointly managed, such as the ones that capture, retain and reuse rainwater [41]. In this regard, in Munich, it is estimated that around 26% of its current rainwater supply could be harvested and then used in food production activities [42]. Considering water harvesting is especially important since droughts in the summer seasons are increasingly frequent and intense in many regions of the planet. Thus, developing models of green structures in UA sites suitable for rainwater harvesting in the rainy months can help maintain the activity during the dry months [17].

The UA benefit most reported by the studies is the food miles. Specht [43] points out that short-distance production-consumption can promote the reuse of organic waste in food production, which avoids higher GHG emissions from the biological waste process, in addition to not generating high emissions due to the low mileage in its distribution perimeter. Thus, it is argued that this can be incorporated as a climate change mitigation system. However, there is still a gap in the assessment of the impact of short-distance production-consumption concerning GHG emissions and other sustainability indicators [43].

Still on emissions, in UA they are affected mainly by the structure, land use, use of fertilizers and the distribution of their production [44]. It is argued that the most significant environmental impact in the food sector is generated by how food is produced, and not necessarily where [44,45], since production and harvesting are responsible for around 83% of GHG emissions from the food sector [46]. In this regard, UA tends to mitigate the environmental impact of the food chain, since in the vast majority of cases it is implemented respecting agroecological and soil regeneration practices and does not have logistic-related emissions [5]. In this sense, Hu et al. [44] in an assessment of GHG emissions from UA and conventional agriculture in Beijing noted that transport was the second most impacting factor on emissions from both modalities; however, in this regard, the UA still had lower emissions compared to conventional agriculture. Therefore, even if the mode of production may not reach an ideal in terms of emissions, UA could still result in a profitable reduction in the emissions of the total sector by dealing with smaller food distribution distances. In addition, UA also eliminates the use of intermediaries in the chain, which results in energy savings [47].

In this sense, Hu et al. [44] propose some possibilities to contain carbon emissions in the activity, such as economic measures granted to urban farmers to encourage the use of solar and wind energy and renewable fuels. However, it is noteworthy to mention that such proposals may not yet be suitable to countries of the Global South, since in the countries of the North the integration of renewable electricity is a topic best addressed in transitions [48]. Although the Global South presents a high potential for renewable energy resources, most of these countries still have limitations in terms of adequate infrastructure for such technologies. It is noteworthy that even with the use of non-renewable energy, in the Global South it is expected that by 2050 around 750 million people will still have no access to electricity [49].

Giraud [45] argues that UA can contribute to the achievement of some Sustainable Development Goals (SDG), such as increasing universal access to renewable energy (SDG#7—”Affordable and Clean Energy”), addressing air pollution in urban areas (SDG#11—Sustainable Cities and Communities) [50], contribute to Climate Action (SDG #13) and support biodiversity (SDG #15—”Life on Land”) [45]. Furthermore, Deksissa et al. [51] argue that UA integration strategies and green rainwater harvesting infrastructure could help urban areas at local and global levels in exploring adaptation mechanisms to extreme climate change events. Likewise, Marçal et al. [52] argue that in the city of Goiânia, UA plays the role of making the urban environment more resilient to the climate crisis by helping mitigate drought, floods, improve soil conservation, capturing carbon from the atmosphere and lowering local air temperature.

However, a limitation presented for the integration of Nature-based Solutions (NBS) policies is that city master plans do not usually consider food resilience in their guidelines [53,54]. As such, the tendency to understand grey and green spaces as separate from each other in urban plans makes the benefits of the synergistic potential that could be achieved by such measures less considered [55,56].

Compared to conventional agriculture, UA is understood to be more resilient due to the shorter production-consumption chain, as well as promoting systems with more diversified production [57]. In this regard, while some studies propose the assessment of sustainability in UA based on life-cycle assessment methodologies [58], other authors argue that these methodologies still underestimate the sustainable and resilient potential of the activity for urban areas [17].

Finally, it is vital to highlight that UA does not necessarily result in the preservation and regeneration of natural resources [59]. For these to happen, it is necessary to develop a transition pathway, which includes technological innovation [59], leading to new models that can maximize ES co-benefits and minimize trade-offs.

### 4.2. The Governance Challenge

The vulnerability of ecosystems and people regarding the climate crisis significantly differ according to the region of the planet, and this is essentially due to factors such as socio-economic development, inequality, marginalization, unsustainable use of land and oceans, as well as governance [60]. The adverse impacts of climate change, development challenges and inequality mutually exacerbate each other. Mortality rates due to floods, droughts, and storms, vary drastically across regions with high and low vulnerability and thus reveal the different starting points in their movement towards climate-resilient development [60].

There is a substantial gap in understanding the political economy of urbanization and the roles of governance [61]. In South and Central America, for example, there are numerous initiatives to improve planning, but they tend to be focused on risk reduction and not on climate adaptation [18]. Thus, there is a gap in the development of planning that promotes adaptation. The solutions lie in improving governance to address global problems, encompassing participation in decision-making, ensuring greater fairness in interventions, as well as creating and improving links with different levels of government and other policies that ensure co-benefits [60].

Although there is evidence regarding the UA contribution to alleviating social and urban challenges, which include climate change, food security, biodiversity and ES, agricultural intensification, resource efficiency, urban renewal and regeneration, land management, public health, social cohesion, and economic growth [62], there is still a gap to link UA as a transformative adaptation measure [63]. It is observed that, in the vast majority of cases, the activity has been driven by community efforts [64] with shallow and unequal public support [53]. Furthermore, UA is often hindered by inadequate planning policies, which discern limited access to land, financial barriers, soil pollution and contamination, development pressure, and gentrification [64]. In addition, finding common ground in different administrative departments in different policy domains involved in UA has not proven easy, at best.

### 4.3. Global North and Global South

Although Clinton et al. [65] have demonstrated from global data that UA has the potential to produce tons of food, sequester tons of nitrogen, save billions of kilowatts of energy, and avoid the loss of billions of cubic meters of rainwater, this type of data is still understood in an academic and impractical way. As a result, the potential multifunctionality offered by UA is not yet engaged in land use planning in many places, which is one of the main obstacles to its adoption, especially in the Global South, where NBS are still timidly implemented [66]. Therefore, understanding UA’s multifunctionality in different geographic contexts is fundamental for its incorporation into urban planning [67].

Some authors postulate that UA presents different approaches between Global North and Global South. First, this division of the world order accepts that the first is composed of countries from North America, Europe, some Asian countries and others located in the southern hemisphere, such as Australia and New Zealand, while the Global South concentrates Latin countries, the African continent, Middle East and parts of Asia. What defines these borders is based on economic, social and political repercussions, representing the division of developed and developing nations [68]. Despite this, the model oversimplifies the characteristics of nations, since it ignores internal variations and commonalities between the Global North and the Global South.

In the context of UA, it is observed that while in the Global North countries these initiatives tend to focus on social dimensions and, in some cases, on environmental benefits, in the Global South, UA is primarily focused on food subsistence and income generation [69,70].

Opitz et al. [70] point out that in the Global South, production generally takes place in polluted environments [71], where health risks prevail due to poor management and environmental pollution [72,73]. In many cases, UA lacks legal status [73] and activities such as leisure or recreation are rarely observed [74,75]. In the Global North, these production spaces tend to have a temporary perspective, since they are threatened by more economically profitable land uses and largely from marketing-oriented initiatives. There is also evidence that populations on a low income, arguably the most nutritionally deprived population, are often excluded from UA [68,70]. Consequently, one of the main challenges reported for the Global North is to make the benefits of UA reach the populations in need, especially in the context of the existence of food deserts in many of these countries [68,70].

The literature understands green infrastructure initiatives related to UA as reflections of specific needs or problems. In Africa, for example, where food security is an important issue, UA is understood as an answer, while in Asia research on green spaces reflects issues of spatial planning and land distribution in high-density cities. In Latin America and the Caribbean, where there are serious issues of inequality, studies highlight vulnerability and social concerns [76].

Another aspect observed is the difference in governmental action in periods of crisis. While in developed countries it is common to observe the encouragement of activities such as UA [53], in developing countries crises can affect even more the performance of governance, as observed in the city of São Paulo during the COVID-19 pandemic [76].

Finally, Dona [67] argues that studies about the potential of UA should be focused on geographical aspects, recognizing the difficulty of establishing a universal pattern of benefits and potentials of the activity, being especially important to promote an evaluation method aimed at developing countries. Further research of UA models encompassing social and environmental aspects simultaneously is needed to understand the potential of this activity under different contexts, especially regarding the diverging Global North and Global South dynamics.

### 4.4. Socio-Environmental Model

Urban food production needs to consider the different dimensions of sustainability; therefore, it must understand the management of environmental challenges, solve or alleviate social problems and promote economic returns [43].

Urban resilience and sustainability depend on the connection between different ES. The provision of ES in cities must seek equality, across socio-economic and demographic regions [77]. The integration of innovations in traditional UA activities, preserving their environmental and social role, can overcome limitations and still offer the chance to achieve the circularity of different resources present in the urban environment [78,79,80].

Accordingly, care must be taken to evaluate the suitability for UA, considering for instance the presence of air pollutants, as well as soil contamination, which expose the health of the local population to risks [43]. The implementation of UA should be directed to places with less exposure to atmospheric and soil contaminants, as well as being based on agroecological production practices to avoid in turn contamination derived from the activity itself.

An aspect not addressed in the literature is to link the implementation of the green structure of food production to the principles of preservation and regeneration of native species. Cities that consider their native vegetation incorporated into municipal land use plans can promote the activity by encouraging and delimiting cultivated species according to the native profile, for instance restricting the use of non-native species in the territory. Cities such as São Paulo encourage this type of action based on municipal plans such as the Atlantic Forest Municipal Plan [81]. Yet, there is no association of this policy with food production in the municipality. Similarly, in the city of Accra, the Land Use and Spatial Planning Act 2016 promotes the protection of different green infrastructures, such as forest reserves and green belts, but does not include UA in its guidelines [79,82]. Thus, the inclusion of UA in policies such as those is an opportunity in potential that needs to be further considered.

Besides, a requalification and assignment of multiple uses of urban green space would address the recommendations of international policies [83] as a fulfillment of Environmental and Urban Agendas [11], such as The New Urban Agenda with the aim to promote a new global model for sustainable urban planning [84]. In this respect, understanding the availability of green areas that can be used in synergy with UA becomes fundamental to avoid the misuse of these spaces and the loss of biodiversity.

The association of green infrastructure and UA is still lacking, even in countries that already adhere to NBS policies. In the case of flood risk management, Deksissa et al. [51] argue that even though their management measures based on green infrastructure can at the same time produce food, and well-planned UA initiatives can contribute to flooding risk management, what happens is that these are policies that are still understood separately. Therefore, the analysis of flood areas may present an opportunity to aggregate UA benefits.

Regarding the economic dimension, UA must be incorporated into commercial chains in the city, preferably in its neighborhood, to contemplate aspects of the food miles. In this regard, Sonnino [85], in an assessment of UA as a local food production system in the Rome region, argues that there is a need for a more effective connection between urban and peri-urban food-producing areas and local commerce, which includes consumers of local fairs. In this way, tracking these potential partnerships can help target locations for food production. Another potential is the connection between producers, as one can direct its production to items that the other producers do not have and vice versa, which not only promotes economic cooperation but also diversifies the availability of products, as well as act as a measure to reduce food waste [85].

The social sphere is perhaps the most fragile to be manipulated since numerous cases of UA implementation are affected by gentrification and mask greenwashing [86,87]. In assessing urban land use for climate adaptation in different cities in the Global South and Global North, Anguelowisk et al. [88] argue that rational and technocratic planning approaches, while defending an ideal “public good”, end up not emphasizing the asymmetrical power dynamics and the conflict over resources present in urban areas [89, 90, 91]. The study points out that in the Global South it is common to see resettlement sites distant from work opportunities, disconnected from social networks and being affected by disaster risks, which reduce the ability of communities to adapt and their long-term security. The implementation of UA must be thought out of the dispute of spaces for rich and poor, being directed in a way equivalent to the needs of each public, as well as preserving the primordial aspect of sustainability, which is to promote environmental and socio-economic development together.

Faced with such challenges, some authors propose strategies for evaluating UA initiatives. Gulya and Edmondson [19] argue that the implementation of UA should be determined according to its scale, the level of integration into the urban fabric, its social inclusion character, the quality of efficiency of food production, as well as social and environmental security promoted. In turn, Tapia et al. [92] developed a model anchored in scientific and sustainability principles, particularly related to SDG and ES narratives, to assess the benefits and negative consequences of implementing different types of UA. Similarly, Zanzi et al. [93] evaluated the sustainability in the implementation of UA by startups in Milan through a holistic framework and observed the possibility of UA to promote four facets of sustainability: economic resilience, social well-being, governance, and environmental integrity. Although relevant, both models are developed for cities belonging to the Global North, whose social and environmental characteristics are very different from those present in the Global South. Besides, the second model is based on the presence of startups, which may present limitations for other realities. Thus, there is a lack of initiatives encompassing the social and environmental particularities observed in southern countries.

Following what has been observed in the literature, this article defends the need to develop an UA assessment model, especially for countries in the Global South, covering socio-economic and environmental criteria. Among the suggested parameters, it is proposed the evaluation of air pollutants, soil contamination, availability of green areas, layout of urban infrastructure (including proximity to users), the profile of native vegetation, locations with potential for capturing rainwater, strategic locations for the circulation of organic waste, commercial establishments with the potential to establish partnerships with producers, as well as local food demand [94, 95, 96, 97].

## 5. Conclusions

The strengthening of sustainable development has promoted the expansion of environmental policies in urban areas, creating opportunities to associate them with food production initiatives, which can not only provide ES to the urban environment but also alleviate social issues related to food and nutrition security. According to the literature, the main obstacle is the association of these different dimensions through solid and active governance, in addition to the fact that it is challenging to stimulate a model for their mainstreaming since the intense socio-economic and environmental particularities of each location imply different implementation needs. In developed countries, UA is not adopted essentially for subsistence, and in some cases it can be a challenge to contemplate populations in food vulnerability, while in developing countries, although very directed towards this population, problems related to local environmental pollution and access to sustainable technologies are often observed in its adoption.

This article defends the need to develop transferable models, in which there is a comprehensive inclusion of the socio-economic and environmental characteristics of each locality.

Environmental parameters are commonly indicated in the development of UA assessment tools, while social ones are still approached in a restricted way, since they can easily be masked or driven by issues of social injustice and gentrification. Therefore, addressing the social aspects of urban environmental policies is arguably one of the main challenges for UA’s success, which also explains why UA is especially complex in the countries of the Global South.

## Figures and Tables

**Table 1:** Selection criteria.

Selection Criteria
-To identify UA models that promote sustainability
-To determine how sustainability is promoted by UA
-To analyse models of UA implementation
-To identify relevant gaps and opportunities about UA implementation

Selection criteria adopted for reading the abstracts of the articles.

**Table 2:** Publications selected for systematic literature review and outcomes categorization.

Authors	Title	Year	Field	Outcome	Continent
Artmann, et al.	The role of urban agriculture as a nature-based solution: a review for developing a systemic assessment framework	2021	Sustainability	Opportunity	Europe
Bellezoni, et al.	Un Understanding and conceptualising how urban green and blue infrastructure affects the food, water, and energy nexus: A synthesis of the literature	2021	Cleaner Production	Gap	South America
Biazoti, et al.	The Impact of COVID-19 on Urban Agriculture inSão Paulo, Brazil	2021	Sustainability	Gap	South America
Canet-Martí, et al.	Nature-Based Solutions for Agriculture in Circular Cities: Challenges, Gaps, and Opportunities	2021	Water	Opportunity	Europe
Deksissa, et al.	Integrating Urban Agriculture and Stormwater Management in a Circular Economy to Enhance Ecosystem Services: Connecting the Dots	2021	Sustainability	Opportunity and Gap	North America
Dobson, et al.	Assessing the Direct Resource Requirements of Urban Horticulture in the United Kingdom: A Citizen Science Approach	2021	Sustainability	Opportunity and Gap	Europe
Dona, et al.	Promoting Urban Agriculture and Its Opportunities and Challenges—A Global Review	2021	Sustainability	Gap	Asia
Dorr, et al.	Environmental impacts and resource use of urban agriculture: a systematic review and meta-analysis	2021	Environment	Gap	Europe
Filippini, et al.	Contribution of periurban farming systems to local food systems: a systemic innovation perspective	2021	Economics	Opportunity and Gap	Europe
Giraud, E.	Urban Food Autonomy: The Flourishing of an Ethics of Care for Sustainability	2021	Humanities	Opportunity	North America
Gómez-Villarino, et al.	Key insights of urban agriculture for sustainable urban development	2021	Agroecology and Sustainable Food Systems	Opportunity	Europe
Gulyas and Edmondson	Increasing City Resilience through Urban Agriculture: Challenges and Solutions in the Global North	2021	Sustainability	Gap	Europe
Hakansson, et al.	Goals and persistence of sustainability experiments in divergent urban contexts: urban agriculture and a geodemographic classification in London	2021	Environment	Gap	Europe
Hu, et al.	Comparative analysis of carbon footprint between conventional smallholder operation and innovative large escale farming of urban agriculture in Beijing, China	2021	Biology	Opportunity	Asia
Langemeyer	Urban agriculture—A necessary pathway towards urban resilience and global sustainability?	2021	Urban Planning	Gap	Europe
Macedo, et al.	Urban green and blue infrastructure: A critical analysis of research on developing countries	2021	Cleaner Production	Gap	South America
Marçal, et al.	UrbaUrban and peri-urban agriculture in Goiania: The search for solutions to adapt cities in the context of global climate change	2021	Urban Climate	Opportunity	South America
Oliveira and Ahmed	Governance of urban agriculture in African cities: Gaps and opportunities for innovation in Accra, Ghana	2021	Cleaner Production	Gap	South America
Opitz, et al.	Contributing to food security in urban areas: differences between urban agriculture and peri-urban agriculture in the Global North	2016	Agriculture and Human Values	Gap	Europe
Orsini, et al.	Urban agriculture in the developing world: A review	2013	Sustainable Development	Opportunity	Europe
Pulighe and Lupia	Food First: COVID-19 Outbreak and Cities Lockdown a Booster for a Wider Vision on Urban Agriculture	2020	Sustainability	Opportunity	Europe
Specht, et al.	Urban agriculture of the future: an overview of sustainability aspects of food production in and on buildings	2014	Agriculture and Human Values	Gap	Europe
Steenkamp, et al.	Food for Thought: Addressing Urban Food Security Risks through Urban Agriculture	2021	Sustainability	Opportunity and Gap	Africa
Tapia, et al.	Monitoring the contribution of urban agriculture to urban sustainability: an indicator-based framework	2021	Sustainable Cities	Opportunity	Europe
Tornaghi	Critical geography of urban agriculture	2014	Geography	Gap	Europe
Yan, et al.	Global Trends in Urban Agriculture Research: A Pathway toward Urban Resilience and Sustainability	2022	Land	Gap	Asia
Zanzi, et al.	Assessing Agri-Food Start-Ups Sustainability in Peri-Urban Agriculture Context	2021	Land	Opportunity	Europe
Zimmerer, et al.	Grand Challenges in Urban Agriculture: Ecological and Social Approaches to Transformative Sustainability	2021	Sustainable Food Systems	Opportunity	North America

List of publications selected categorized by outcome.

## Data Availability

Not applicable.
